# Supplementary material for: AI cancer driver mutation predictions are valid in real-world data
Source: Nat Commun. 2025 Sep 26;16:8509. doi: 10.1038/s41467-025-63461-8 (PMC12474978; doi:10.1038/s41467-025-63461-8)
Supplement: Supplementary file 1 — Supplementary Information [file 41467_2025_63461_MOESM1_ESM.pdf]

Supplementary Appendix for “AI cancer driver mutation predictions are valid in real-world data”

Supplementary Figures

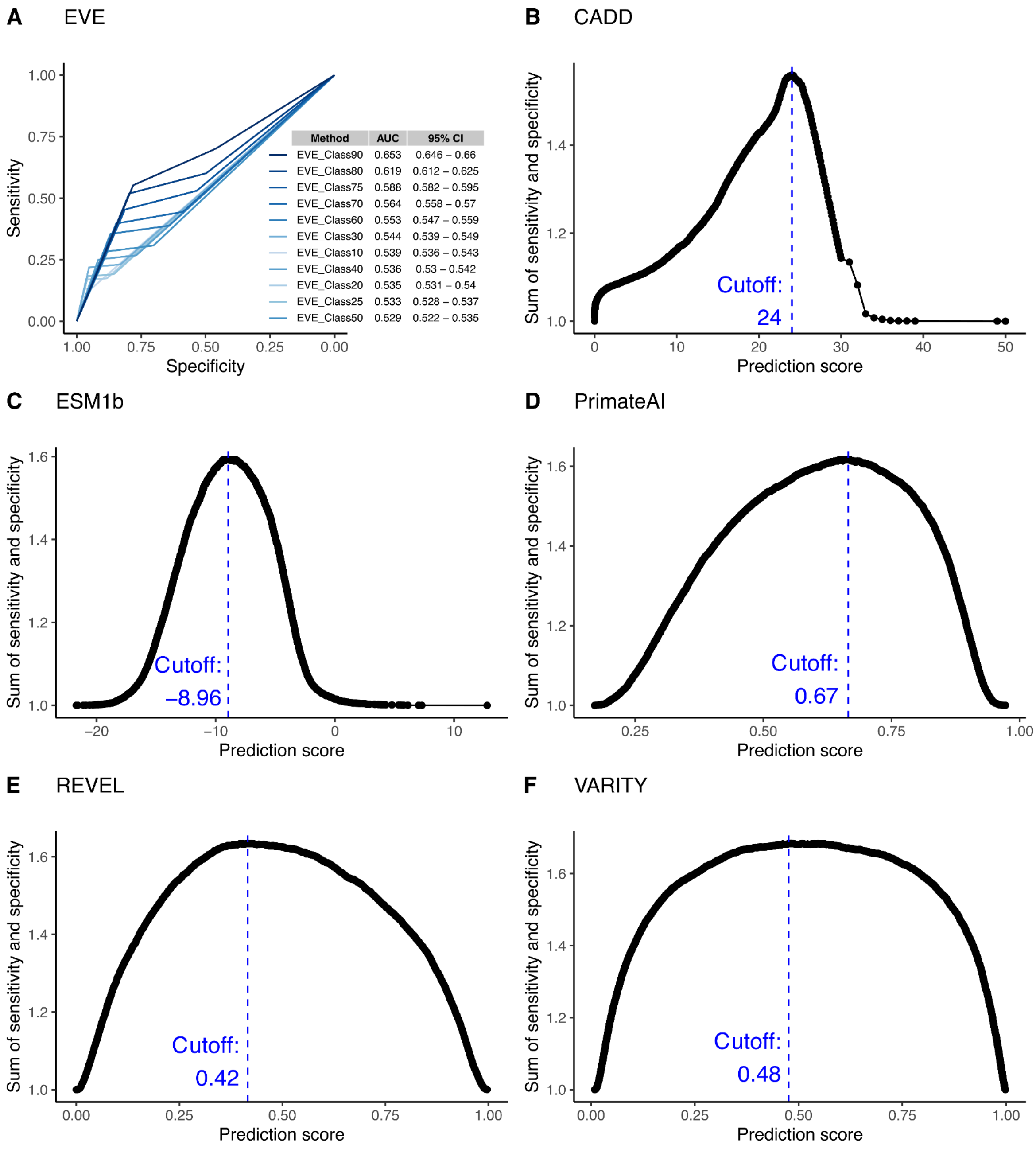

Figure S1. Determination of score cutoffs for pathogenic mutations

The score cutoff thresholds for pathogenic mutations for methods without off-the-shelf classification were determined by optimizing the sum of sensitivity and specificity in classifying oncogenic mutations and non-oncogenic dbSNPs. Source data are provided as a Source Data file.

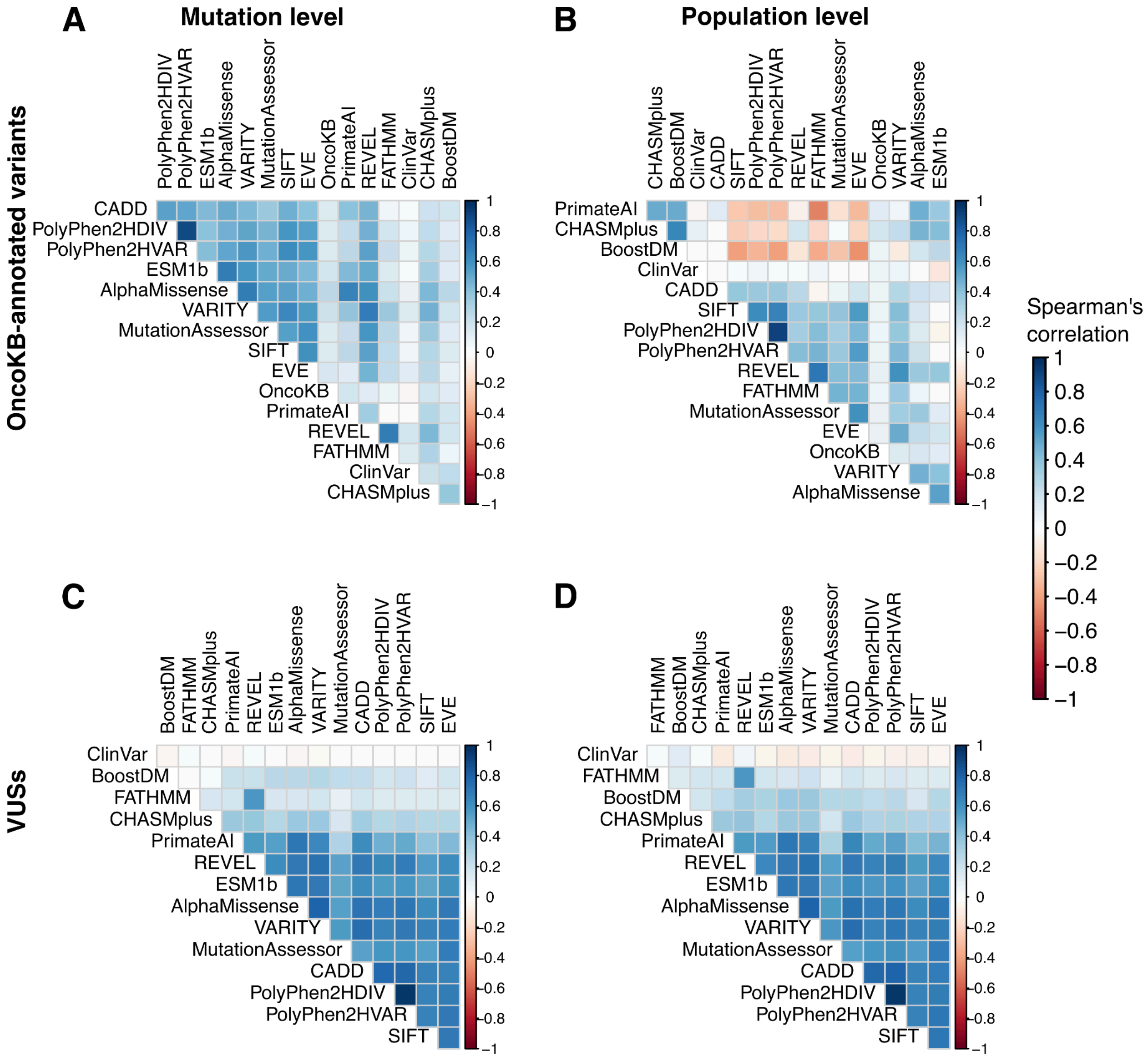

Figure S2. Concordance heatmap of mutation annotations GENIE

Spearman's correlation was used to calculate the correlation between the predicted classification by each method (non-pathogenic or pathogenic, as per Supplemental Methods) and as annotated by OncoKB (unknown or oncogenic). Tile colors depict Spearman's correlation coefficients. Source data are provided as a Source Data file.

A. Correlation at the mutation level, in which each unique mutation is included once. OncoKB-annotated variants N=8,033, VUSs N=503,530.

B. Correlation at the population level, in which all occurrences of missense mutations are included. OncoKB-annotated variants N=180,540, VUSs N=925,376.

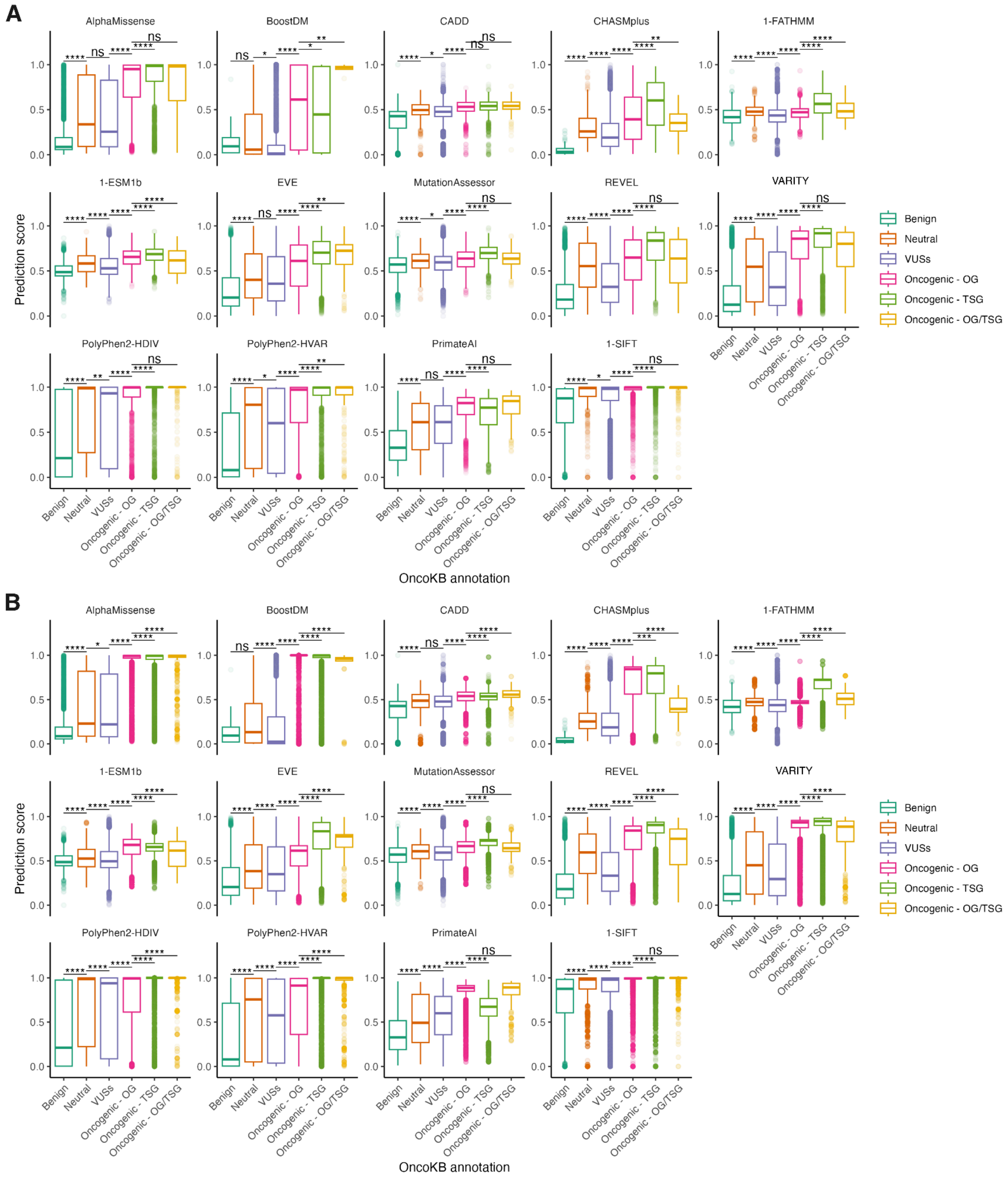

Figure S3. Prediction scores of missense mutations and benign dbSNPs

Distribution of prediction scores from benign dbSNPs and missense mutations in GENIE v.14-public, broken down by their occurrence in oncogenes (OG), tumor suppressor genes (TSG) or genes that act as both (OG/TSG). For most VEPs, scores are in the range of 0-1, with higher scores suggest higher predicted pathogenicity. For ease of comparison, scores from

methods with different ranges, including CADD, MutationAssessor, FATHMM and ESM1b, were scaled to the 0-1 range. For methods where lower scores indicated higher pathogenicity, the difference between 1 and the (scaled) prediction scores was plotted. Boxplots depict means +- interquartile ranges.

Brackets depict significance in two-sided Tukey's range test for pairwise difference in means with FDR correction. *: q-value ≤ 0.05, **: q-value ≤ 0.01, ***: q-value ≤ 0.001, ****: q-value ≤ 1e-04.

A. Score distributions at the mutation level, in which each unique mutation is included once. Benign dbSNPs N=7,474, GENIE v14 mutations in oncogenes (OG, N=140,309), tumor suppressor genes (TSG, N=222,668) or genes that act as both (OG/TSG, N=29,657).
B. Score distributions at the population level, in which all occurrences of missense mutations are included. Benign dbSNPs N=7,474, GENIE v14 mutations in oncogenes (OG, N=408,771), tumor suppressor genes (TSG, N=506,068) or genes that act as both (OG/TSG, N=57,592).

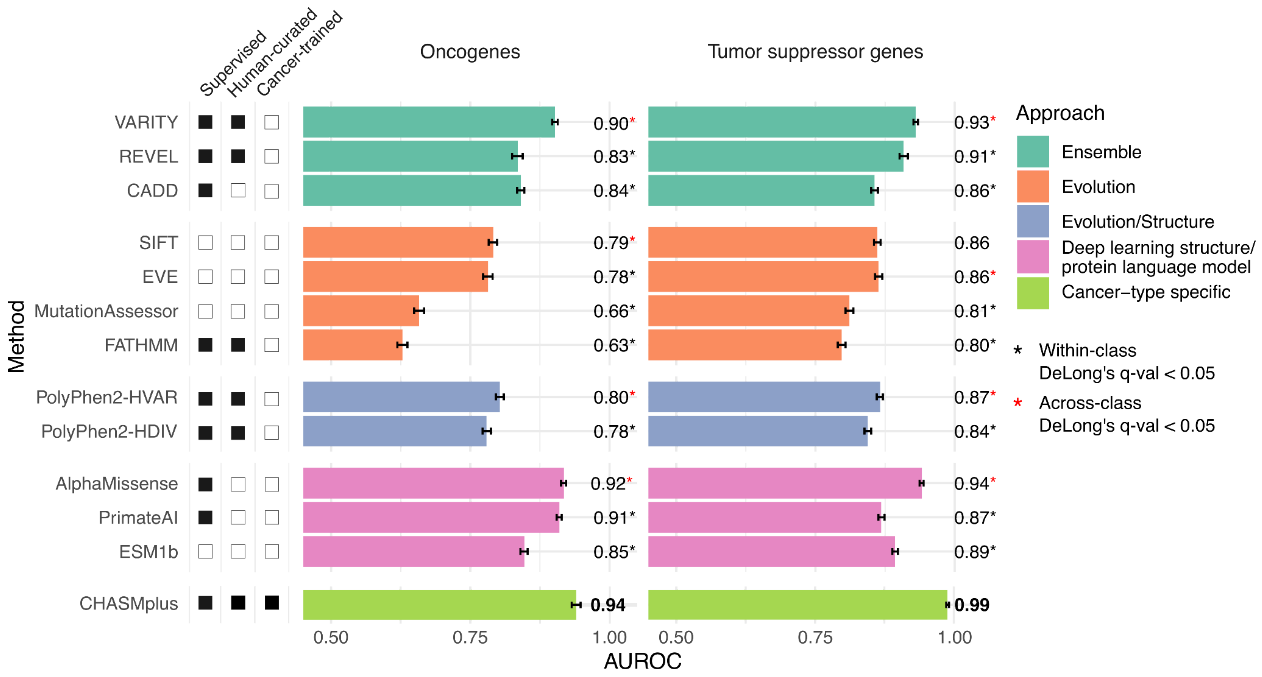

Figure S4. Mutation-level evaluation of VEP performance in identifying pathogenic cancer mutations.

AUROCs (± 95%CI) of 13 variant annotation methods in classifying known oncogenic mutations (N=8,033) and non-oncogenic SNPs (N=7,474 upsampled from 7,474) at the population level. DeLong's test was used to compare AUROCs with FDR correction. Within each methodological class, pairwise comparisons were performed between the top-performing method and others (*: q ≤ 0.05 marked by black asterisks). Red asterisks denote significant differences (q ≤ 0.05) between each class's top performer and the overall best method (bolded AUROC). Source data are provided as a Source Data file.

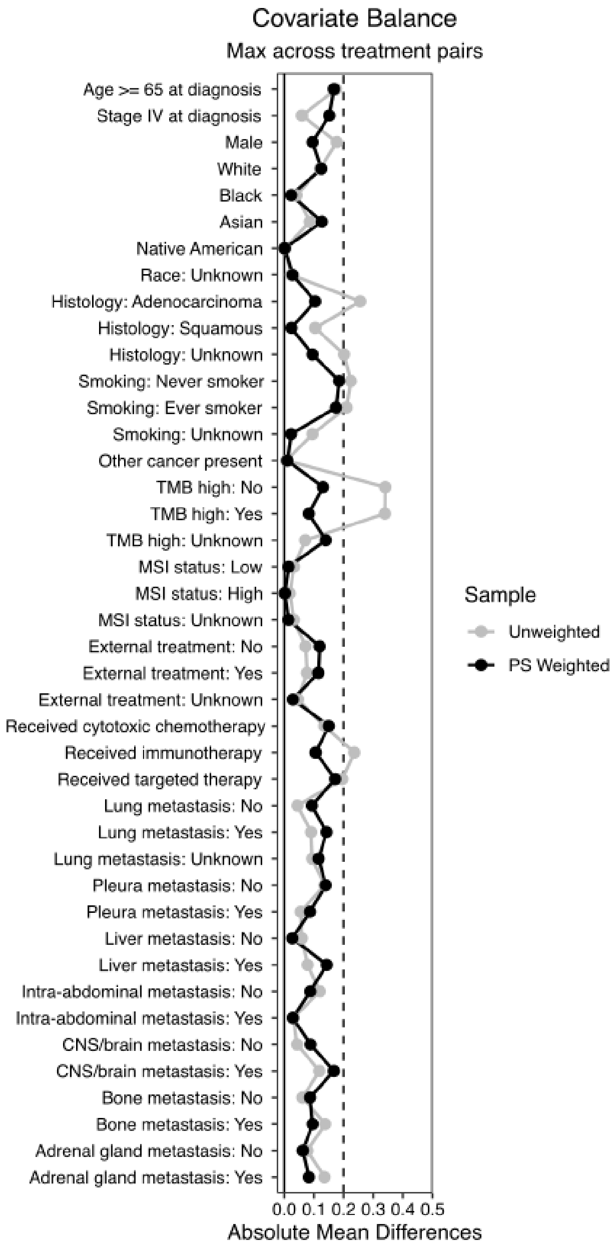

Figure S5. Balance of covariates between patient strata using inverse probability of treatment scores

Absolute mean differences between clinical and demographic covariates in four groups of patients stratified by KEAP1 mutational status, including No KEAP1 mutation, KEAP1 - Oncogenic mutations, KEAP1 - reclassified pathogenic mutations by AlphaMissense, KEAP1 - Reclassified benign mutations by AlphaMissense before and after inverse probability of treatment score weighting (IPTW). IPTW helps achieve balance in variables with large imbalance between groups, such as TMB high status. Statistics derived from N=7965 MSK-IMPACT NSCLC patients. Source data are provided as a Source Data file.

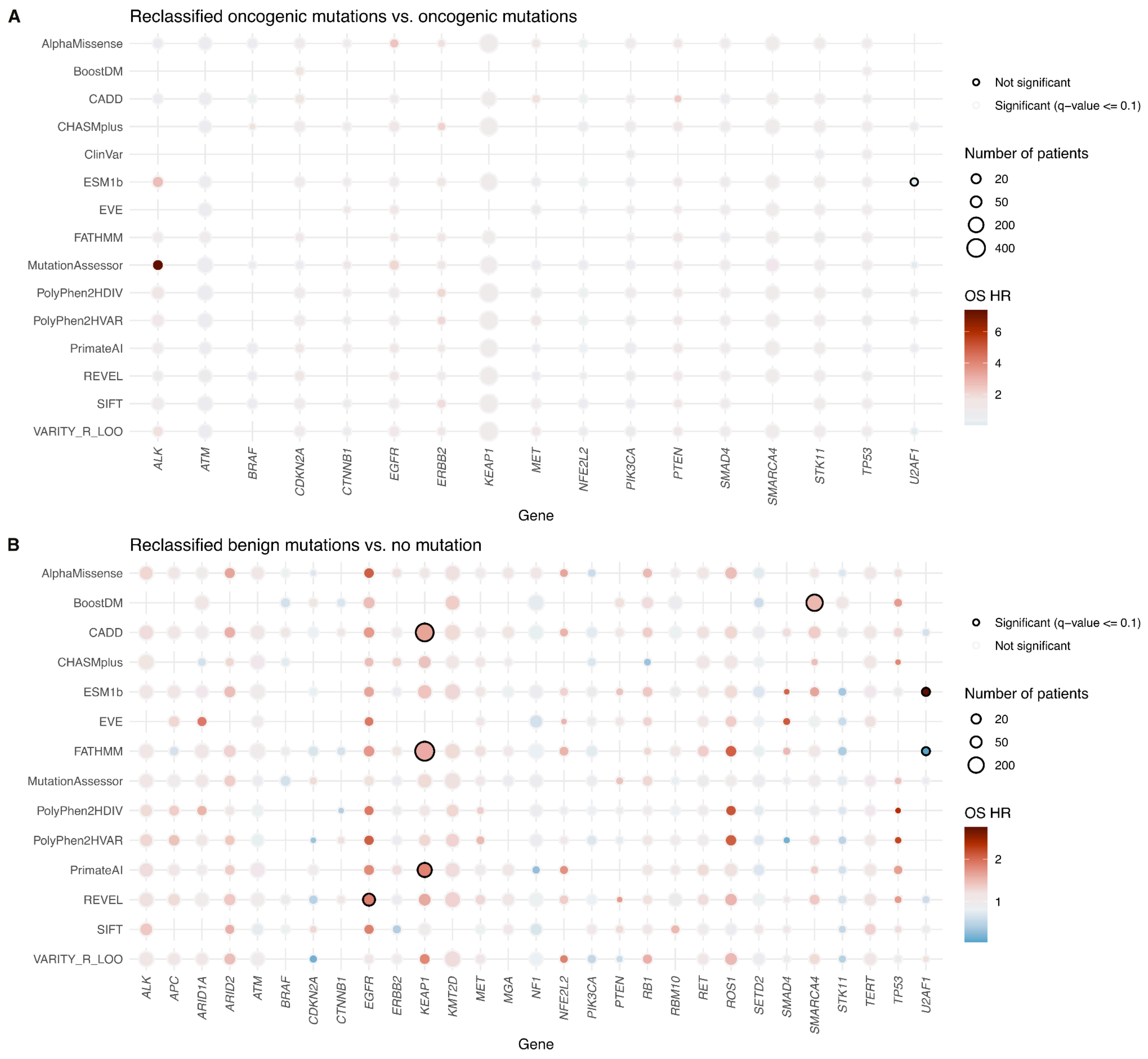

Figure S6. Cox proportional hazards coefficients of reclassified mutations versus known oncogenic mutations or no mutation

Statistics derived from analyses with MSK-IMPACT NSCLC (N=7,965 patients) cohort. Source data are provided as a Source Data file.

A. Overall survival hazard ratios of patients with reclassified pathogenic mutations compared to patients with oncogenic mutations were obtained from weighted Cox proportional hazard models.

B. Overall survival hazard ratios of patients with reclassified benign mutations compared to patients with no mutation in a given gene were obtained from weighted Cox proportional hazard models.

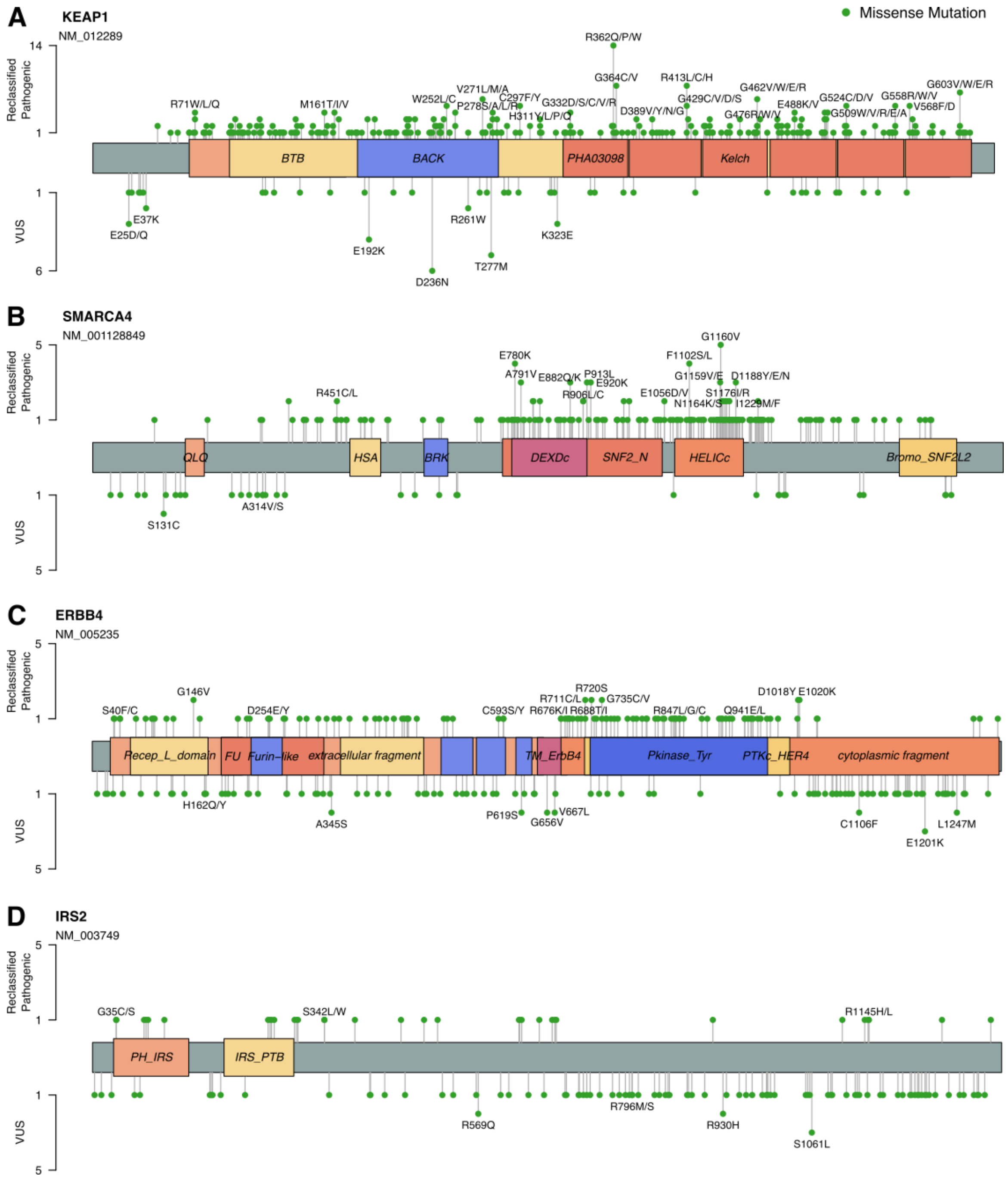

Figure S7. Lollipop plots of VUSs reclassified as pathogenic or benign by AlphaMissense

Amino acid-level changes resulting from AlphaMissense-reclassified pathogenic mutations in in the MSK-IMPACT NSCLC cohort are plotted for:

A. *KEAP1*
B. *SMARCA4*
C. *ERBB4*
D. *IRS2*

The most commonly mutated residues are labeled. Source data are provided as a Source Data file.

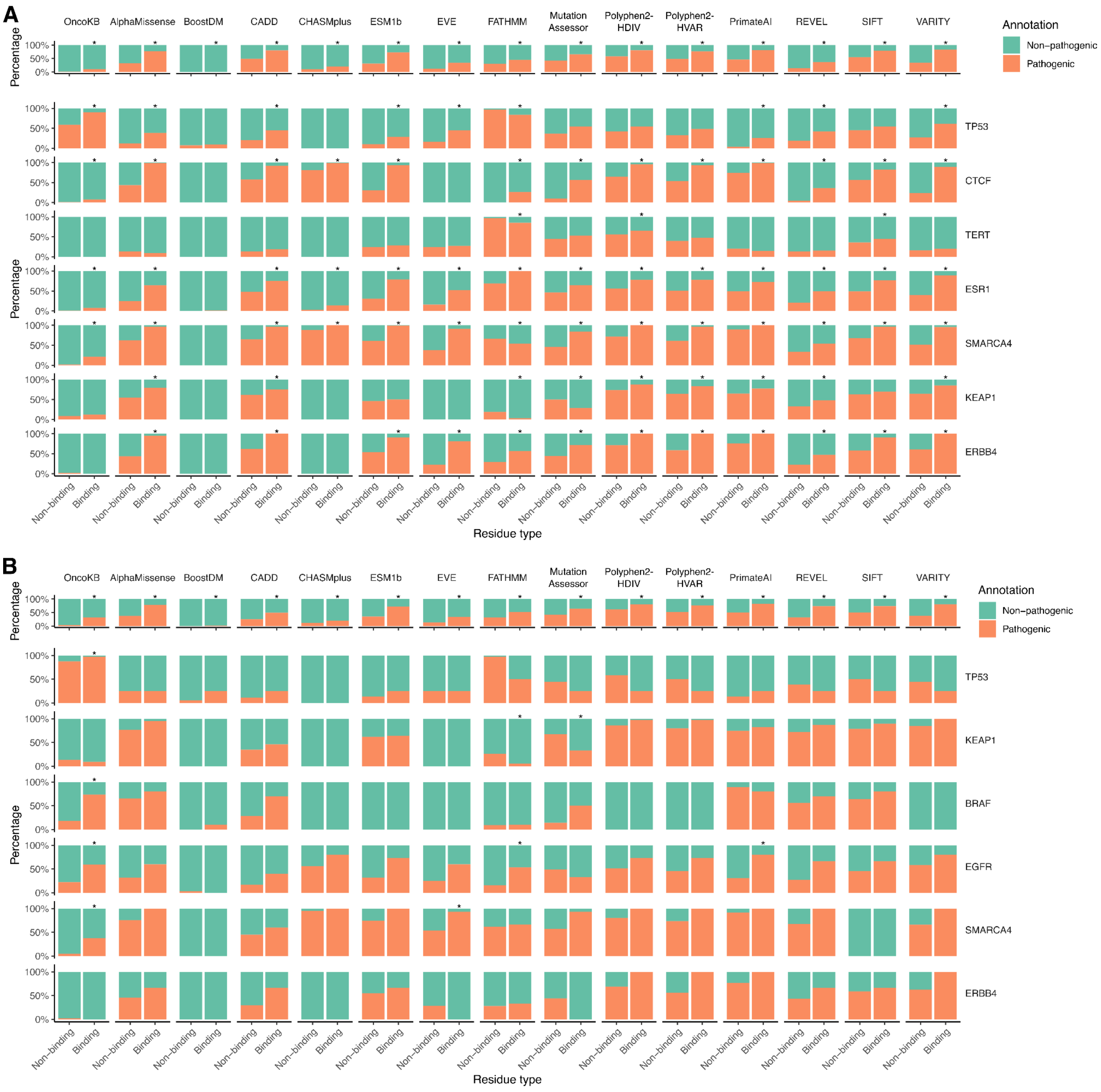

Figure S8. Reclassified pathogenic mutations occur at ligand-binding residues and protein-protein interaction (PPI) hotspots

Frequency and annotation of missense mutations occurring at binding residues (either ligand binding or PPI hotspots, see Supplemental Appendix) or non-binding residues of genes with high number of binding residues in **A.** GENIE v14-public (N=209,588) and **B.** MSK-IMPACT NSCLC cohort (N=35,260).

*: q-value ≤ 0.1, two-sided Fisher's exact test with FDR correction. Source data are provided as a Source Data file.

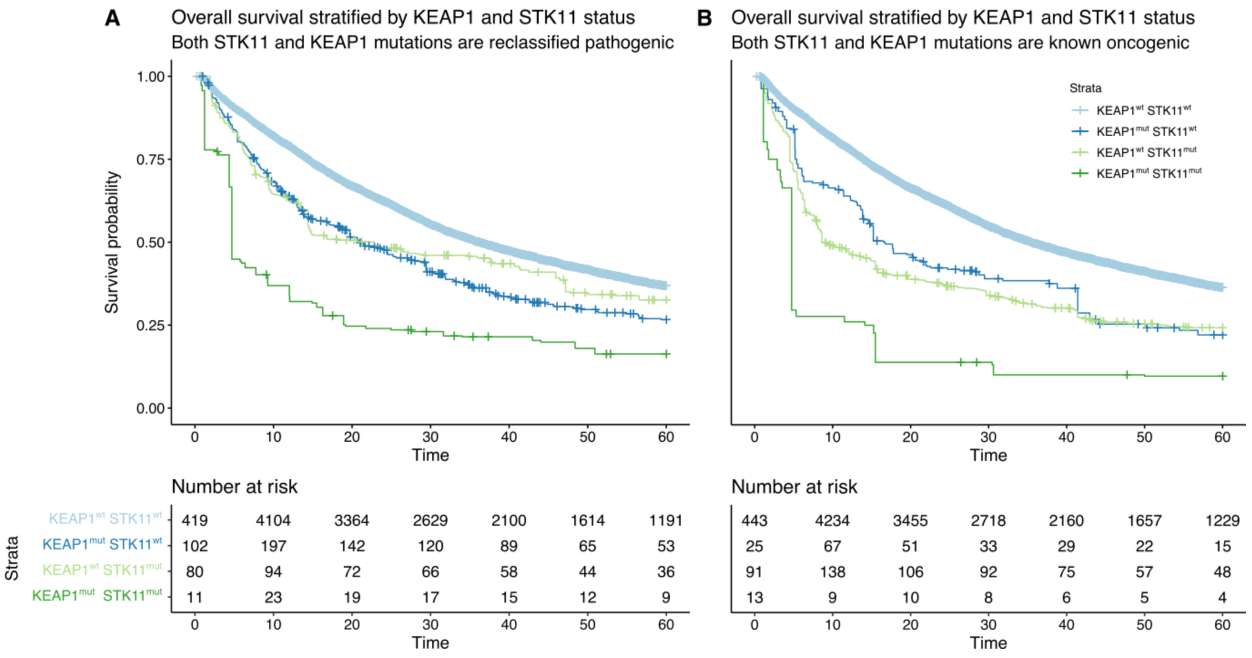

Figure S9. Outcomes of patients with concurrent *STK11* and *KEAP1* mutations
Analyses were done on MSK-IMPACT NSCLC cohort (N=7,695 patients). Source data are provided as a Source Data file.

A. Kaplan-Meier curves comparing patients with concurrent reclassified pathogenic *KEAP1* and *STK11* mutations versus reclassified pathogenic mutations in either gene or without any mutations.

B. Kaplan-Meier curves comparing patients with concurrent OncoKB oncogenic *KEAP1* and *STK11* mutations versus oncogenic mutations in either gene or without any mutations.

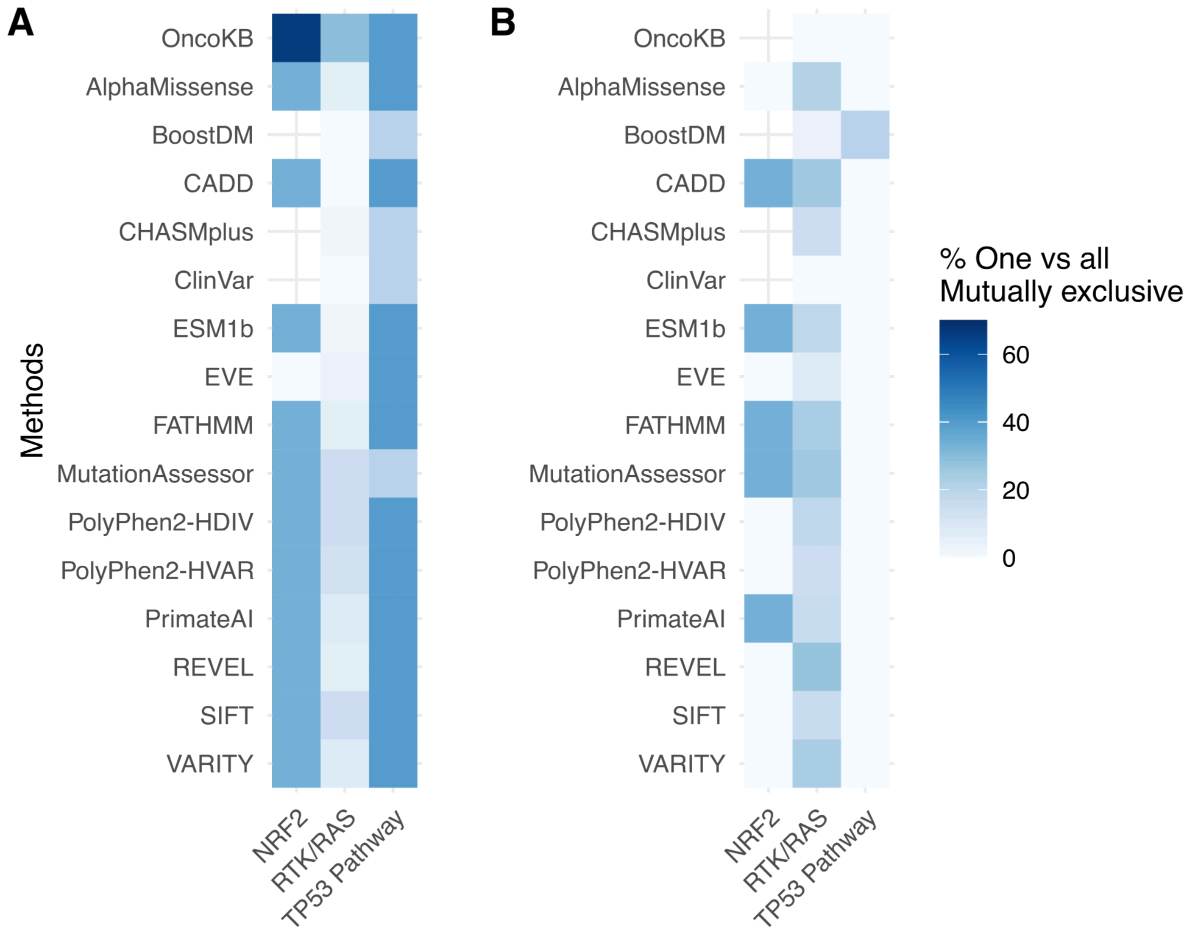

Figure S10. Mutual exclusivity of mutations in oncogenic signaling pathways

One-versus-all mutual exclusivity for each gene in a pathway is determined using a two-sided Fisher's exact test. Mutual exclusivity percent for each pathway is then calculated as the number of FDR-corrected significant tests divided by the total number of tests. Analyses were performed on MSK-IMPACT NSCLC cohort, N=7,695. Source data are provided as a Source Data file.

A. One-versus-all mutual exclusivity between reclassified pathogenic mutations of each gene and known oncogenic mutations in all other genes in a given pathway.

B. One-versus-all mutual exclusivity between reclassified benign mutations/VUSs of each gene and known oncogenic mutations in all other genes in a given pathway.

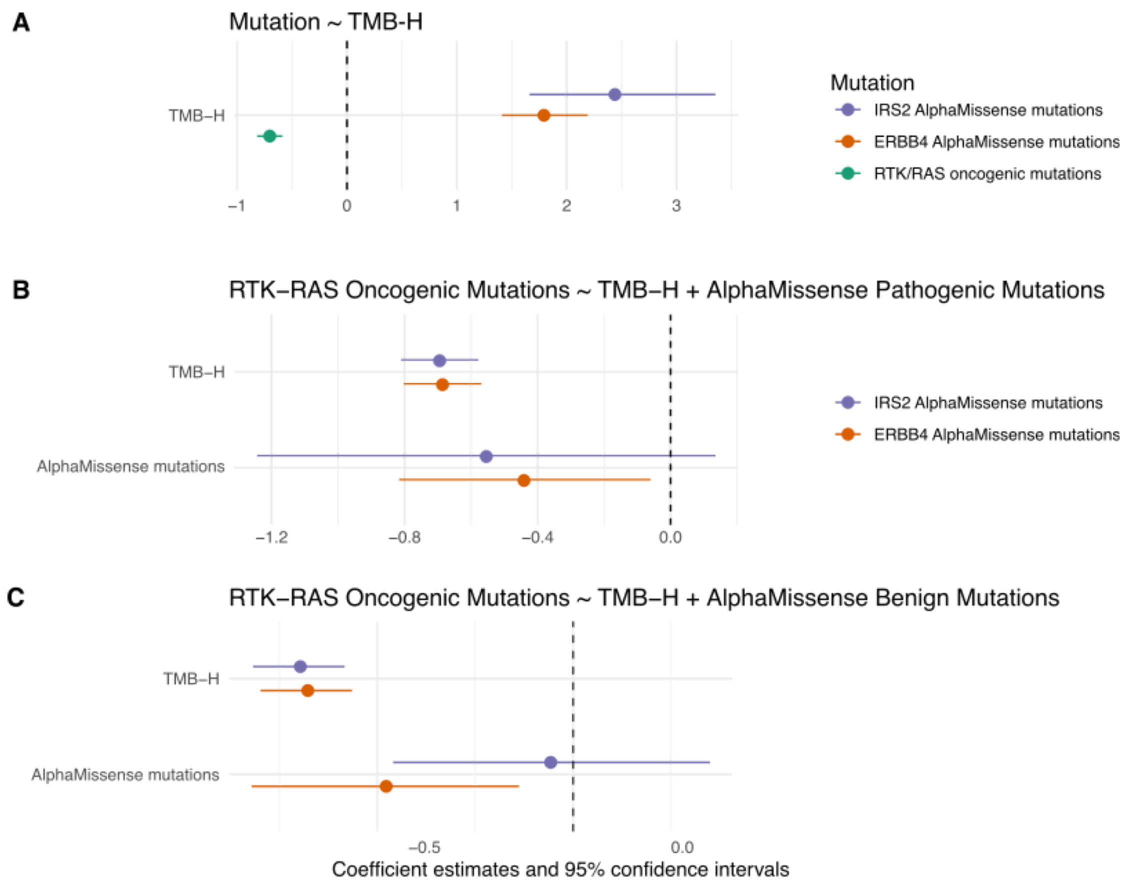

Figure S11. Mutations in *ERBB4* and *IRS2* are mutually exclusive with RTK/RAS oncogenic mutations independent of TMB-H status

All regressions were performed with a cohort of N=6,743 MSK-IMPACT NSCLC patients with mutations in the RTK/RAS pathway.

A. Coefficients for TMB-H status regressed on either RTK/RAS oncogenic mutations or AlphaMissense reclassified mutations in *ERBB4* and *IRS2*. AlphaMissense reclassified pathogenic mutations in *ERBB4* and *IRS2* are positively correlated with TMB-H status, suggesting they occur more frequently in TMB-H samples, whereas RTK/RAS known oncogenic mutations are observed frequently in TMB-low samples.
B. Coefficients for TMB-H status and AlphaMissense pathogenic mutations in either *IRS2* or *ERBB4* regressed on RTK/RAS oncogenic mutations.
C. Coefficients for TMB-H status and AlphaMissense benign mutations in either *IRS2* or *ERBB4* regressed on RTK/RAS oncogenic mutations.

All AlphaMissense mutations in *ERBB4* are significantly and negatively associated with RTK/RAS oncogenic mutations independently from TMB-H status, while all AlphaMissense mutations in *IRS2* are trending towards a negative association with RTK/RAS oncogenic mutations, although small sample sizes in both genes preclude definitive conclusion.

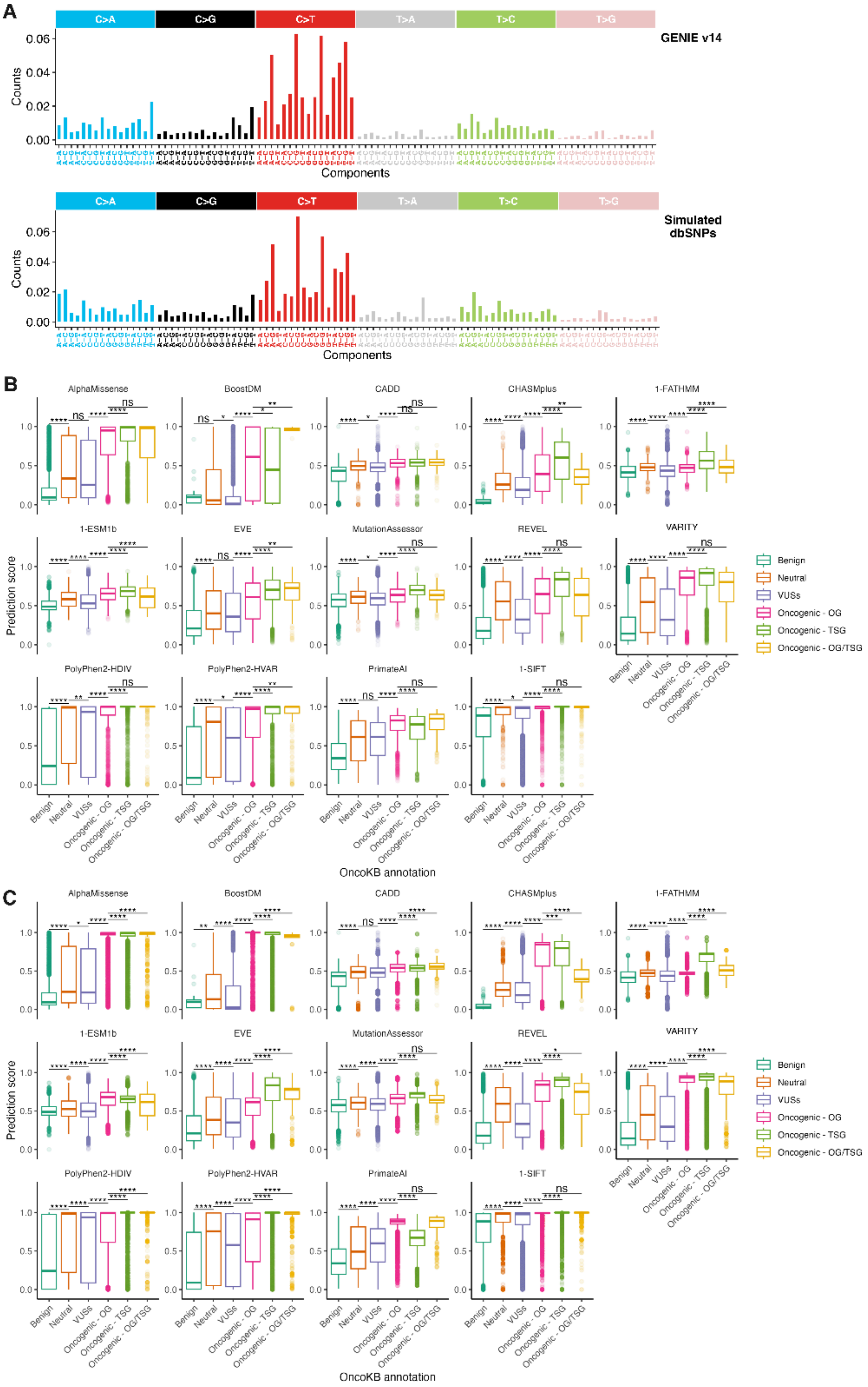
A
C>A
C>G
C>T
T>A
T>C
T>G
GENIE v14
Counts
Components
Simulated dbSNPs
B
AlphaMissense
BoostDM
CADD
CHASMplus
1-FATHMM
1-ESM1b
EVE
MutationAssessor
REVEL
VARITY
PolyPhen2-HDIV
PolyPhen2-HVAR
PrimateAI
1-SIFT
Prediction score
OncoKB annotation
Benign
Neutral
VUSs
Oncogenic - OG
Oncogenic - TSG
Oncogenic - OG/TSG
C
AlphaMissense
BoostDM
CADD
CHASMplus
1-FATHMM
1-ESM1b
EVE
MutationAssessor
REVEL
VARITY
PolyPhen2-HDIV
PolyPhen2-HVAR
PrimateAI
1-SIFT
Prediction score
OncoKB annotation
Benign
Neutral
VUSs
Oncogenic - OG
Oncogenic - TSG
Oncogenic - OG/TSG

Figure S12. Prediction scores of missense mutations and simulated benign mutations based on tri-nucleotides mutational probability

We sampled dbSNP from mutational signatures to generate a set of benign mutations with similar mutational compositions as observed in GENIE v14, then used this simulated set of mutations as benign mutations in our comparison.

Distribution of prediction scores from simulated benign mutations (N=20,000) and missense mutations in GENIE v.14-public, broken down by their occurrence in oncogenes (OG), tumor suppressor genes (TSG) or genes that act as both (OG/TSG). For most VEPs, scores are in the range of 0-1, with higher scores suggest higher predicted pathogenicity. For ease of comparison, scores from methods with different ranges, including CADD, MutationAssessor, FATHMM and ESM1b, were scaled to the 0-1 range. For methods where lower scores indicated higher pathogenicity, the difference between 1 and the (scaled) prediction scores was plotted. Boxplots depict means +- interquartile ranges (IQR), with whiskers extending +/- 1.5 IQR).

Brackets depict significance in two-sided Tukey's range test for pairwise difference in means with FDR correction. *: q-value ≤ 0.05, **: q-value ≤ 0.01, ***: q-value ≤ 0.001, ****: q-value ≤ 1e-04.

Source data are provided as a Source Data file.

A. Distributions of mutation types in the GENIE v14 cohort (top) and in the simulated dbSNPs set.
B. Score distributions at the mutation level, in which each unique mutation is included once. Benign dbSNPs N=20,000, GENIE v14 mutations in oncogenes (OG, N=140,309), tumor suppressor genes (TSG, N=222,668) or genes that act as both (OG/TSG, N=29,657).
C. Score distributions at the population level, in which all occurrences of missense mutations are included. Simulated dbSNPs N=20,000, GENIE v14 mutations in oncogenes (OG, N=408,771), tumor suppressor genes (TSG, N=506,068) or genes that act as both (OG/TSG, N=57,592).

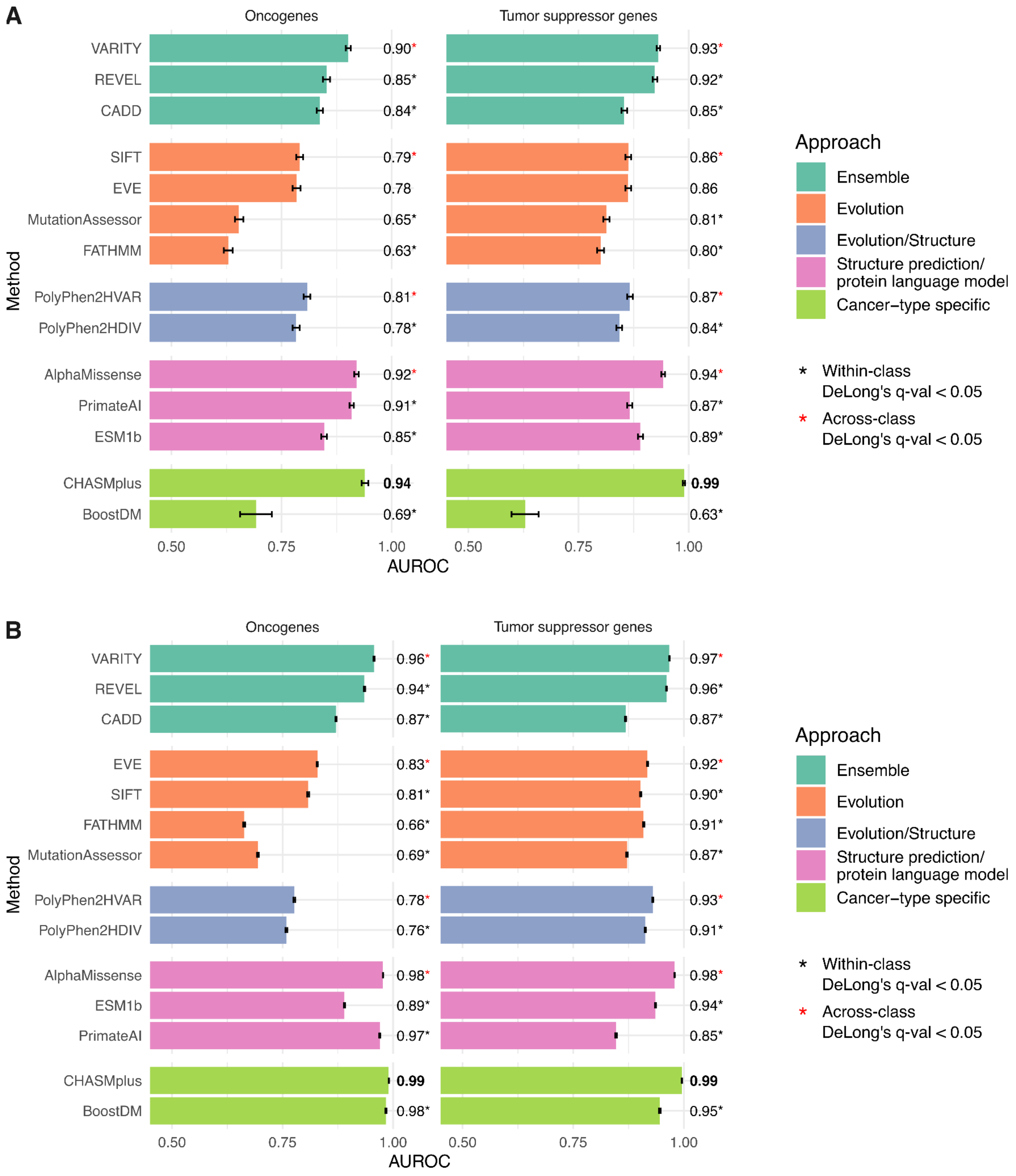

Figure S13. AUROC of VEPs in classifying missense mutations and simulated benign mutations based on tri-nucleotides mutational probability

Areas under the receiver operating curves (AUROC) showing performance of 14 variant annotation methods in classifying known oncogenic mutations and non-oncogenic SNPs at the

A. mutation level, where each unique mutation is counted once. Benign dbSNPs N=10,000 downsampled from 20,000, GENIE v14 mutations known oncogenic mutations N=8,033.
B. population level, where each occurrence of a mutation is counted once. Simulated dbSNPs N=20,000, GENIE v14 known oncogenic mutations N=180,540.

Source data are provided as a Source Data file.

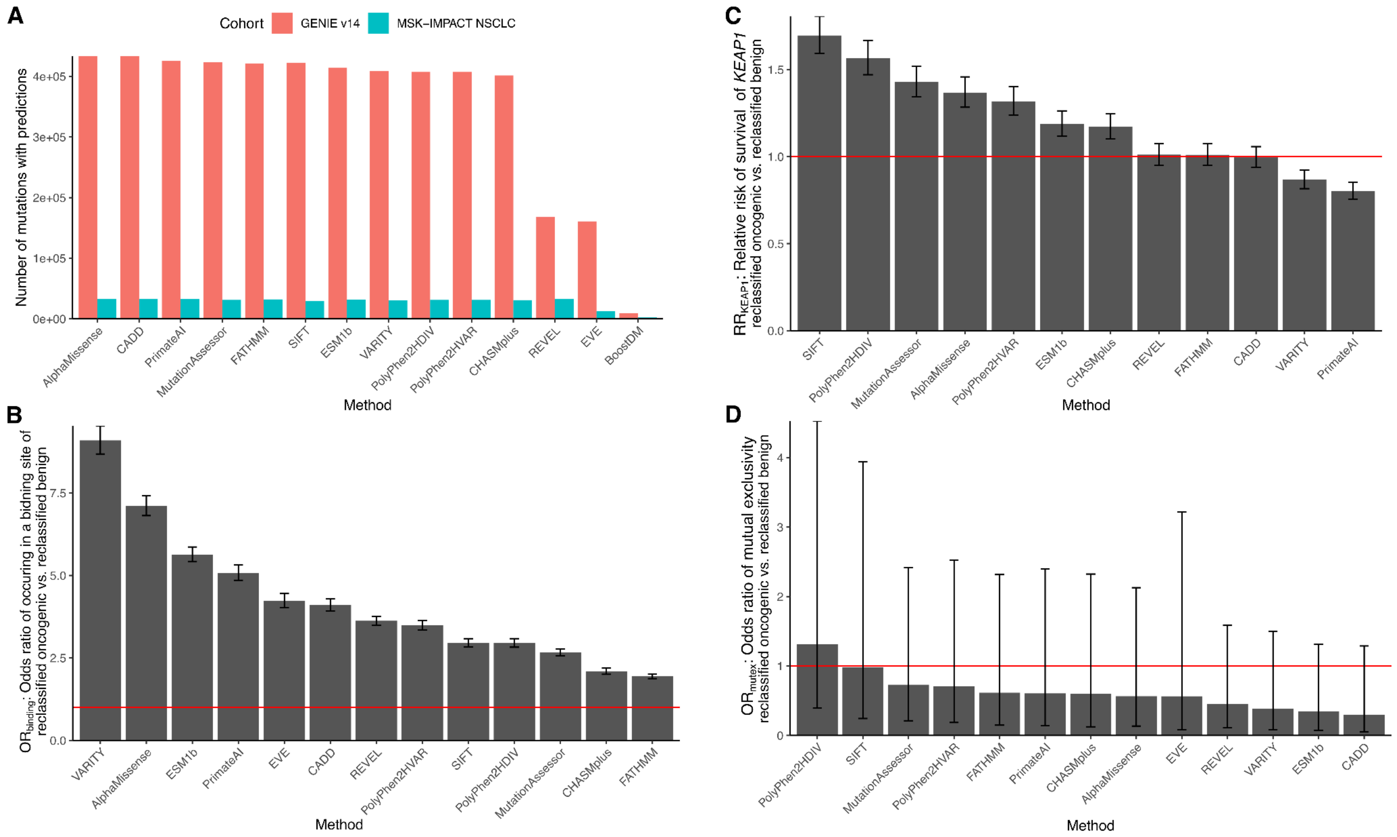

Figure S14. Performance metrics of VEPs across three validation domains

A. Number of mutations with predictions by each method in GENIE v14 and MSK-IMPACT NSCLC cohorts.
B. The odds ratio (OR) compares the likelihood of reclassified oncogenic mutations versus reclassified benign mutations occurring at protein binding sites with available crystal structures. This metric was calculated using the GENIE v14 cohort (same data presented in Figure 2A). For OR > 1 (red line), a larger OR indicates that oncogenic mutations are more likely to occur at binding sites than benign mutations, reflecting a method's ability to better distinguish pathogenic from benign mutations.
C. The relative risk of survival is the ratio of overall survival (OS) hazard ratios (HR) for patients with *KEAP1* reclassified oncogenic mutations versus those with no *KEAP1* mutation, compared to the HR for patients with *KEAP1* reclassified benign mutations versus no *KEAP1* mutation. Relative risk and 95% confidence intervals were calculated as per Tunes da Silva et al. (2009)[21] using MSK-IMPACT NSCLC data (as shown in Figures 2B and S6B). A relative risk greater than 1 (red line) indicates that patients with oncogenic KEAP1 mutations have significantly worse OS than those with benign mutations. A higher relative risk suggests better distinction between potentially pathogenic mutations affecting outcomes and other VUSs.
D. OR of reclassified oncogenic mutations, compared to reclassified benign mutations, being mutually exclusive with other oncogenic mutations across three oncogenic

pathways in NSCLC. This metric was calculated using MSK-IMPACT NSCLC data (same data presented in Figure 2C). OR > 1 (red line) suggests that oncogenic mutations are more likely to be mutually exclusive with other known oncogenic mutations within the same pathway, indicating the method's effectiveness in identifying potential driver mutations.

Source data are provided as a Source Data file.

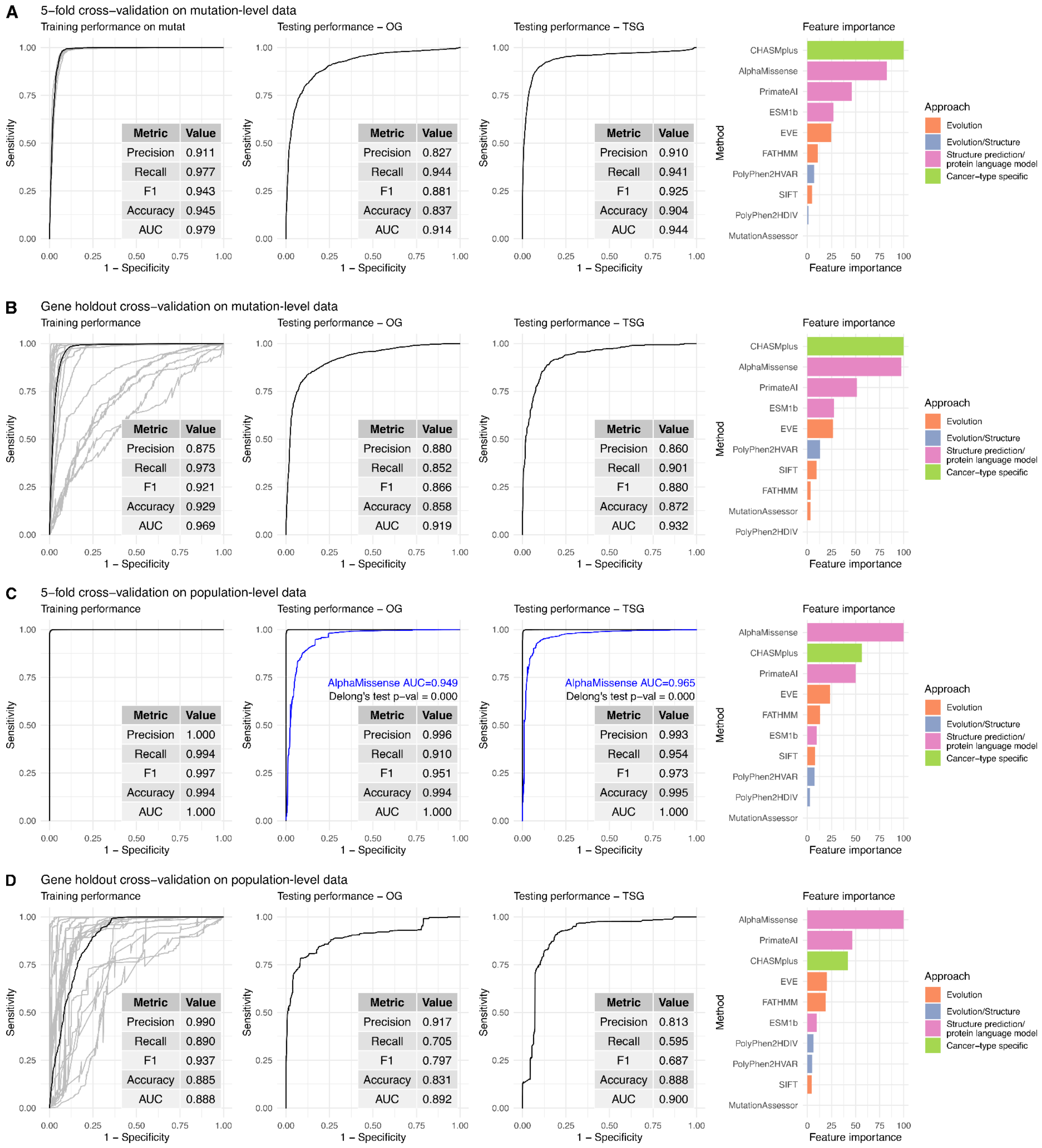

Figure S15. Performance metrics of random forest ensemble for variant pathogenicity prediction

Random forest ensembles were trained using predictions from 10 non-ensemble methods examined in this study to classify variant pathogenicity. OncoKB oncogenic variants in GENIE v14 served as the positive class, while randomly selected variants from dbSNP constituted the

negative class. The dataset was split into a 75:25 ratio for training and testing. Models were either trained on **A-B.** mutation level data, which only counts each unique mutation once (N=7,474 non-oncogenic, N=8,033 oncogenic), and **C-D.** population level data, which counts all occurrences of mutations in GENIE v14 (N=180,540 non-oncogenic upsampled from 7,474 unique non-oncogenic mutations, N=180,540 known oncogenic). The models were evaluated using two approaches**: A, C.** random 5-fold cross-validation and **B, D.** gene holdout cross-validation. We further compared the performance of the best performing ensemble, which is trained on population-level data and validated using 5-fold cross-validation (**C**) with the best performing non-ensemble method AlphaMissense using Delong's test.
Source data are provided as a Source Data file.

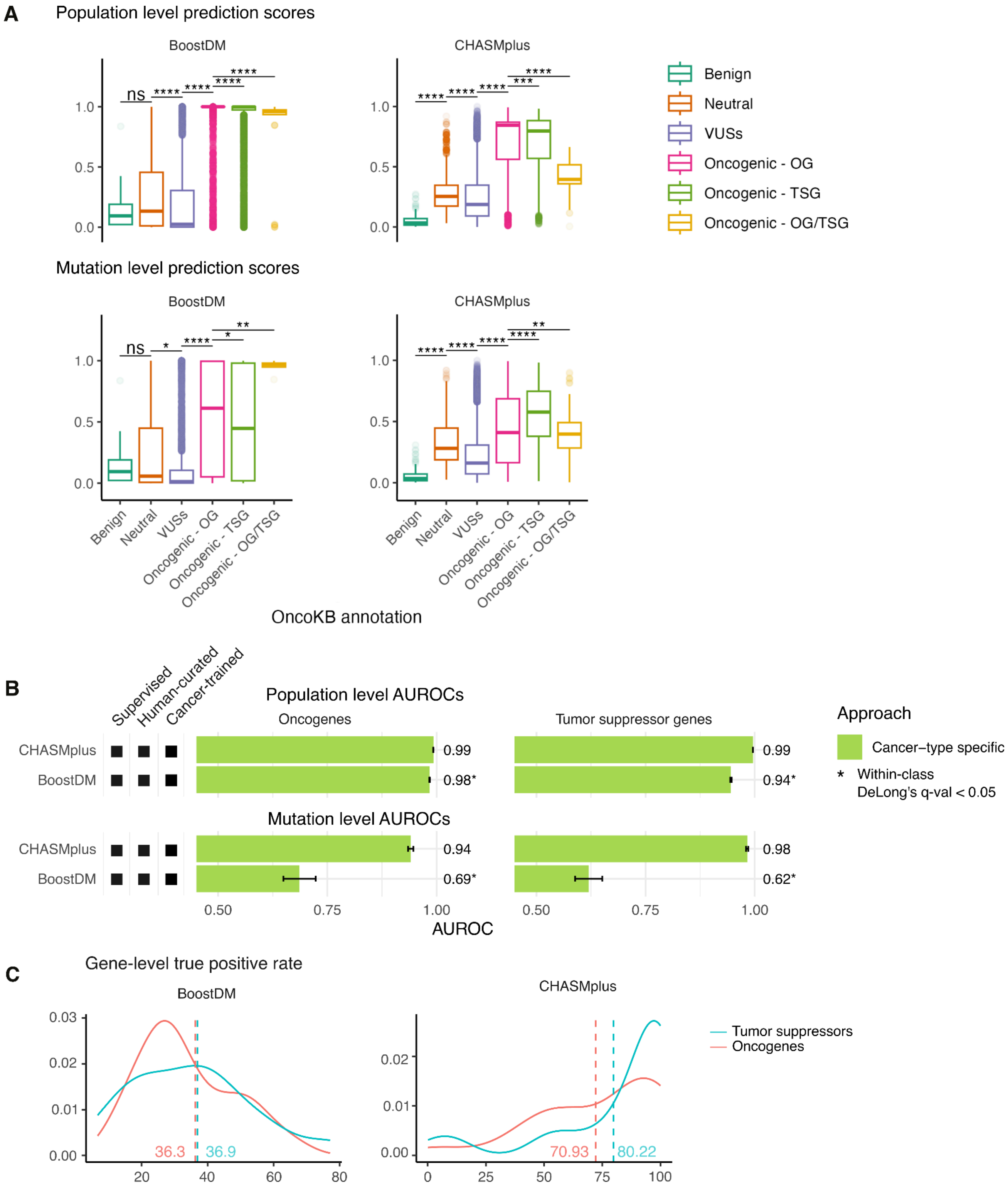

Figure S16. Cancer type-specific methods performance in annotating known oncogenic variants

Since BoostDM and CHASMplus are trained on cancer recurrence data, which other generalist methods are not and thus could lead to an unfair comparison, we conducted separate analyses to evaluate their performance. All analyses were performed on GENIE v14 data.

A. Distributions of prediction scores of BoostDM and CHASMplus from 7,474 non-pathogenic dbSNPs and missense mutations in GENIE v.14-public, broken down by their occurrence in oncogenes (OG), tumor suppressor genes (TSG) or genes that act as both (OG/TSG) at the population level, in which all occurrences of missense mutations are included (**left**), and at the mutation-level, in which each unique mutation is only counted once (**right**). Points higher on the y-axis corresponded with higher predicted pathogenicity. Boxplots depict means +- interquartile ranges. Brackets depict significance in two-sided Tukey's range test with FDR correction. *: q-value <= 0.05, **: q-value <= 0.01, ****: q-value <= 1e-4.
B. Bar chart showing AUROC (+/-95%CI) of two cancer-type specific VEPs, CHASMplus and BoostDM, in classifying known oncogenic mutations and non-oncogenic SNPs at the population level (**top**) and mutation level (**bottom**). Significant differences between AUROCs were tested using DeLong's tests and p-values were corrected for multiple hypothesis testing.
C. Density plots showing true positive rates (TPR) of BoostDM and CHASMplus over all genes. TPR is defined as the number of known oncogenic mutations accurately annotated as pathogenic by BoostDM divided by the total number of known oncogenic mutations. See Supplemental Appendix for a complete list of TPRs.

Source data are provided as a Source Data file.

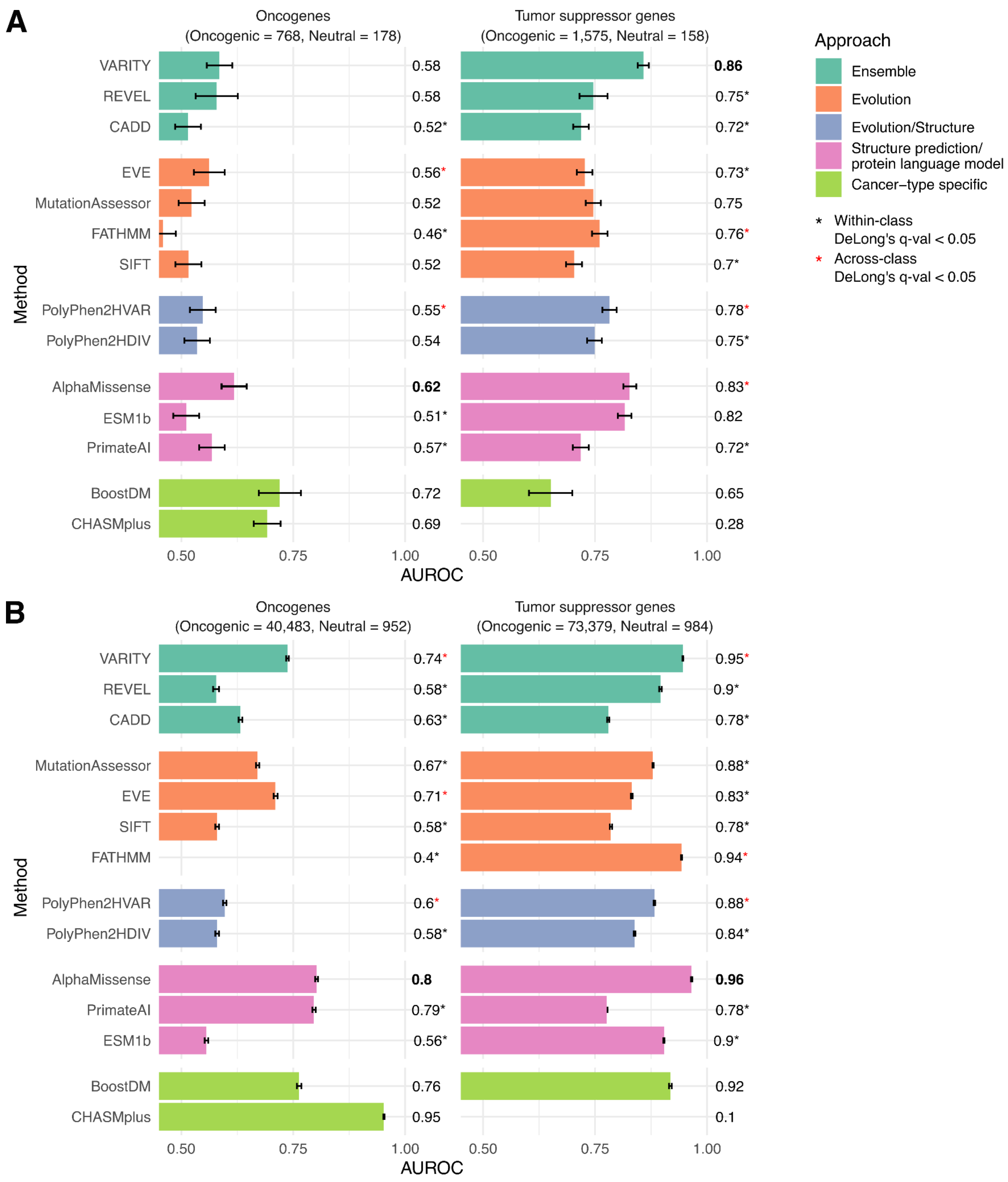

Figure S17. AUROC of VEPs in classifying missense oncogenic mutations and neutral mutations

Areas under the receiver operating curves (AUROC) showing performance of 14 variant annotation methods in classifying known oncogenic mutations and known neutral mutations, as annotated by OncoKB. Only genes with at least 10 neutral and 10 oncogenic mutations are included in this analysis.

Significant differences between AUROCs were tested using DeLong's tests for all methods except for cancer-specific methods, as these methods were trained on recurrence information that the rest did not have access to. P-values were corrected for multiple hypothesis testing. Within each class of approach, pairwise comparisons were carried out between the best-performing method and all other methods of the same class (significant differences denoted with black asterisks). Pairwise comparisons between the best-performing method of each approach and the best overall method (denoted by bold AUC) were also carried out, with significant differences denoted by red asterisks.

A. AUROC (+/-95%CI) calculated at mutation level, in which each unique mutation was included once. GENIE v14 mutations in oncogenes (oncogenic N=768, neutral N=178) and tumor suppressor genes (oncogenic N=1,575, neutral N=158) are included.
B. AUROC (+/-95%CI) calculated at population level, in which each occurrence of a mutation was included. GENIE v14 mutations in oncogenes (oncogenic N=40,483, neutral N=952) and tumor suppressor genes (oncogenic N=73,379, neutral N=984) are included.

Source data are provided as a Source Data file.

Supplementary Tables

Table S1. Annotation methods overview. Six variant function prediction methods and two databases were evaluated in this study.

**Method**	**Description**	**Required human-curated data**
AlphaMissense	Deep neural network based on the protein structure predictor AlphaFold2, trained on weak labels derived from human and primate germline variants	No
BoostDM	Gene-tumor-specific gradient boosting classifiers trained on observed mutations in tumors and simulated benign mutations.	Yes
CADD	Support vector machine model combining conservation metrics and functional genomic data, trained on weak labels derived from human germline data variants	No
CHASMplus	Semi-supervised cancer-type-specific models incorporating 95 features including mutation hotspots, evolutionary conservation, molecular function annotations, sequence-biased regions, and gene-level covariates.	Yes
ClinVar	Database of human variants and supporting evidences for their functions	Yes
ESM1b	Unsupervised deep protein language model	No
EVE	Unsupervised deep learning model based on distribution of sequence variation across organisms	No
FATHMM	Variant effect predictor based on sequence conservation within hidden Markov models, weighted for cancer-associated mutations	Yes
MutationAssessor	Variant functional impact predictor based on evolutionary conservation patterns across protein families and subfamilies	No

OncoKB	FDA-approved somatic mutation database	Yes
Polyphen2-HDIV	Damaging mutation classifier based on 11 sequence, homology and structural features, trained on Mendelian disease variants and closely related homologs	Yes
Polyphen2-HVAR	Damaging mutation classifier based on 11 sequence, homology and structural features, trained on disease-causing mutations and non-disease-related nsSNPs	Yes
PrimateAI	Deep learning network, incorporating information from two sub-networks that predict secondary structure and solvent accessibility, trained on common human variants and primate variation	No
REVEL	Random forest-based ensemble method trained on other prediction methods and curated variants	Yes
SIFT	Sequence homology-based prediction of amino acid substitution	No
VARITY (VARITY_R_LOO score)	XGBoost model incorporating scores from unsupervised VEPs as well as information about protein-protein interaction and protein structure. VARITY_R_LOO is the VARITY model trained on rare ClinVar variants (MAF < 0.5%) and cross-validated using a leave-one-variant-out strategy	Yes

Table S2. VEP prediction score cutoffs for variant classifications

	**GENIE v14**	**MSK-IMPACT NSCLC**	**Non-MSK GENIE BPC NSCLC**
EVE	Class90	Class90	Class90
CADD	24	24	23.6
ESM1b	-8.96	-9.11	-4.3
PrimateAI	0.67	0.67	0.6
REVEL	0.42	0.42	0.44

VARITY_R_LOO	0.48	0.48	0.66

Table S3. Count and proportion of mutations in GENIE v.14-public grouped by their predicted functions and OncoKB annotations at the mutation level, where each unique mutation is counted once.

Method/ Database	Method Annotation	OncoKB: Unknown (N=503,530)		OncoKB: Neutral (N=677)		OncoKB: Oncogenic (N=7,466)		OncoKB: Resistance (N=81)	
		# of mutations	% of total	# of mutations	% of total	# of mutations	% of total	# of mutations	% of total
AlphaMissense	Non-pathogenic	275,229	52.85	336	0.06	1,015	0.19	6	0
	Pathogenic	166,463	31.97	269	0.05	5,944	1.14	72	0.01
	Unknown	70,234	13.49	87	0.02	1,074	0.21	3	0
CADD	Non-pathogenic	249,836	47.98	275	0	1,332	0.26	7	0
	Pathogenic	252,528	48.49	401	0	6,110	1.17	73	0.01
	Unknown	9,562	1.84	16	0	591	0.11	1	0
ClinVar	Non-pathogenic	2,998	0.58	75	0.01	28	0.01	0	0
	Pathogenic	2,504	0.48	110	0.02	1,425	0.27	12	0
	Unknown	506,424	97.25	507	0.1	6,580	1.26	69	0.01
ESM1b	Non-pathogenic	312520	61.07	293	0.06	1742	0.34	17	0
	Pathogenic	163705	31.99	267	0.05	5410	1.06	61	0.01
	Unknown	27305	5.34	117	0.02	314	0.06	3	0
EVE	Non-pathogenic	104315	20.38	204	0.04	1143	0.22	10	0
	Pathogenic	65599	12.82	155	0.03	3554	0.69	31	0.01
	Unknown	333616	65.19	318	0.06	2769	0.54	40	0.01
FATHMM	Non-pathogenic	332501	64.97	337	0.07	2973	0.58	28	0.01
	Pathogenic	152804	29.86	327	0.06	4315	0.84	51	0.01
	Unknown	18225	3.56	13	0	178	0.03	2	0
Mutation Assessor	Non-pathogenic	269492	52.66	326	0.06	1978	0.39	51	0.01
	Pathogenic	213919	41.8	341	0.07	5289	1.03	30	0.01

	Unknown	20119	3.93	10	0	199	0.04	0	0
PolyPhen2HDIV	Non-pathogenic	173569	33.92	176	0.03	877	0.17	9	0
	Pathogenic	294375	57.52	380	0.07	6248	1.22	69	0.01
	Unknown	35586	6.95	121	0.02	341	0.07	3	0
PolyPhen2HVAR	Non-pathogenic	222851	43.55	242	0.05	1247	0.24	10	0
	Pathogenic	245093	47.89	314	0.06	5878	1.15	68	0.01
	Unknown	35586	6.95	121	0.02	341	0.07	3	0
PrimateAI	Non-pathogenic	254821	49.79	338	0.07	1703	0.33	4	0
	Pathogenic	236114	46.14	337	0.07	5733	1.12	77	0.02
	Unknown	12595	2.46	2	0	30	0.01	0	0
REVEL	Non-pathogenic	132056	25.8	66	0.01	654	0.13	5	0
	Pathogenic	58637	11.46	88	0.02	1481	0.29	27	0.01
	Unknown	312837	61.13	523	0.1	5331	1.04	49	0.01
SIFT	Non-pathogenic	202790	39.63	243	0.05	1151	0.22	17	0
	Pathogenic	283145	55.33	419	0.08	6066	1.19	62	0.01
	Unknown	17595	3.44	15	0	249	0.05	2	0
VARITY_R_LOO	Non-pathogenic	290183	56.7	252	0.05	1009	0.2	9	0
	Pathogenic	177613	34.71	308	0.06	6110	1.19	69	0.01
	Unknown	35734	6.98	117	0.02	347	0.07	3	0

Table S4. Count and proportion of mutations in GENIE v.14-public grouped by their predicted functions and OncoKB annotations at the population level, where all occurrences of mutations in the dataset are counted.

Method/ Database	Method Annotation	OncoKB: Unknown (N=911,113)		OncoKB: Neutral (N=4,102)		OncoKB: Oncogenic (N=180,540)		OncoKB: Resistance (N=515)	
		# of mutations	% of total	# of mutations	% of total	# of mutations	% of total	# of mutations	% of total
AlphaMissense	Non-pathogenic	531,225	44.63	2,277	0.19	9,105	0.76	23	0
	Pathogenic	285,513	23.99	1,493	0.13	223,898	18.81	675	0.06

	Unknown	122,141	10.26	477	0.04	13,398	1.13	9	0
CADD	Non-patho genic	464,410	39.02	1,951	0.16	26,116	2.19	16	0
	Pathogenic	460,696	38.71	2,249	0.19	216,378	18.18	685	0.06
	Unknown	13,773	1.16	47	0	3,907	0.33	6	0
ClinVar	Non-patho genic	30,103	2.53	717	0.06	323	0.03	0	0
	Pathogenic	18,643	1.57	1,071	0.09	145,246	12.2	241	0.02
	Unknown	890,133	74.79	2,459	0.21	100,832	8.47	466	0.04
ESM1b	Non-patho genic	574,574	52.41	2,062	0.19	30,278	2.76	96	0.01
	Pathogenic	286,721	26.15	1,426	0.13	141,208	12.88	403	0.04
	Unknown	49,818	4.54	614	0.06	9,054	0.83	16	0
EVE	Non-patho genic	198,217	18.08	1,317	0.12	26,819	2.45	153	0.01
	Pathogenic	119,842	10.93	899	0.08	99,835	9.11	208	0.02
	Unknown	593,054	54.1	1,886	0.17	53,886	4.92	154	0.01
FATHMM	Non-patho genic	597,977	54.55	2,247	0.2	77,068	7.03	178	0.02
	Pathogenic	280,271	25.57	1,793	0.16	94,545	8.62	327	0.03
	Unknown	32,865	3	62	0.01	8,927	0.81	10	0
Mutation Assessor	Non-patho genic	496,686	45.31	2,130	0.19	46,565	4.25	368	0.03
	Pathogenic	377,468	34.43	1,924	0.18	130,641	11.92	147	0.01
	Unknown	36,959	3.37	48	0	3,334	0.3	0	0
PolyPhen 2HDIV	Non-patho genic	317491	28.96	1179	0.11	24030	2.19	23	0
	Pathogenic	530150	48.36	2309	0.21	147294	13.44	476	0.04
	Unknown	63472	5.79	614	0.06	9216	0.84	16	0
PolyPhen 2HVAR	Non-patho genic	412450	37.62	1590	0.15	33303	3.04	28	0
	Pathogenic	435191	39.7	1898	0.17	138021	12.59	471	0.04
	Unknown	63472	5.79	614	0.06	9216	0.84	16	0
PrimateAI	Non-patho genic	476039	43.42	2324	0.21	31795	2.9	13	0
	Pathogenic	411014	37.49	1766	0.16	148620	13.56	502	0.05
	Unknown	24060	2.19	12	0	125	0.01	0	0

REVEL	Non-pathogenic	231694	21.13	592	0.05	13135	1.2	27	0
	Pathogenic	110298	10.06	757	0.07	69650	6.35	282	0.03
	Unknown	569121	51.91	2753	0.25	97755	8.92	206	0.02
SIFT	Non-pathogenic	376741	34.37	1626	0.15	10836	0.99	172	0.02
	Pathogenic	501043	45.7	2411	0.22	160597	14.65	333	0.03
	Unknown	33329	3.04	65	0.01	9107	0.83	10	0
VARITY_R_LOO	Non-pathogenic	543488	49.58	1834	0.17	12415	1.13	29	0
	Pathogenic	303799	27.71	1654	0.15	157370	14.36	470	0.04
	Unknown	63826	5.82	614	0.06	10755	0.98	16	0

Table S5. Population characteristics of patients in the MSK-IMPACT cohort with non-small cell lung cancer (NSCLC) included in this study.

	**MSK-IMPACT NSCLC** (N=7,965)
**SEX**	
MALE	3287 (41.3%)
FEMALE	4678 (58.7%)
**AGE AT DIAGNOSIS**	
< 65	3364 (42.2%)
>= 65	4601 (57.8%)
**RACE SELF-REPORTED**	
WHITE/CAUCASIAN	5135 (64.5%)
EAST ASIAN	519 (6.5%)
AFRICAN	236 (3.0%)
SOUTH ASIAN	80 (1.0%)
NATIVE AMERICAN	13 (0.2%)
OTHER	539 (6.8%)
MISSING	1443 (18.1%)
**EVER SMOKING**	
NO	1795 (22.5%)
YES	4825 (60.6%)
MISSING	1345 (16.9%)

**RECEIVED PRIOR CHEMOTHERAPY**	
NO	3324 (41.7%)
YES	4641 (58.3%)
**RECEIVED PRIOR TARGETED THERAPY**	
NO	4993 (62.7%)
YES	2972 (37.3%)
**RECEIVED PRIOR IMMUNOTHERAPY**	
NO	5107 (64.1%)
YES	2858 (35.9%)

References

1. Tran, T. N. *et al.* Abstract 4259: Identification of anti-neoplastic therapy given before initial visit at a referral center using natural language processing applied to medical oncology initial consultation notes. *Cancer Res.* **83**, 4259–4259 (2023).
2. Jee, J. *et al.* Abstract 5721: Automated annotation for large-scale clinicogenomic models of lung cancer treatment response and overall survival. *Cancer Res.* **83**, 5721–5721 (2023).
3. Luthra, A. *et al.* Abstract 1158: A.I.-assisted clinical data curation to determine genomic biomarkers of cancer metastasis. *Cancer Res.* **82**, 1158–1158 (2022).
4. Do, R. K. G. *et al.* Patterns of Metastatic Disease in Patients with Cancer Derived from Natural Language Processing of Structured CT Radiology Reports over a 10-year Period. *Radiology* **301**, 115–122 (2021).
5. Middha, S. *et al.* Reliable Pan-Cancer Microsatellite Instability Assessment by Using Targeted Next-Generation Sequencing Data. *JCO Precis. Oncol.* **2017**, (2017).
6. Pugh, T. J. *et al.* AACR project GENIE: 100,000 cases and beyond. *Cancer Discov.* **12**, 2044–2057 (2022).
7. Choudhury, N. J. *et al.* The GENIE BPC NSCLC Cohort: A Real-World Repository Integrating Standardized Clinical and Genomic Data for 1,846 Patients with Non-Small Cell Lung Cancer. *Clin. Cancer Res.* **29**, 3418–3428 (2023).
8. Wang, D., Li, J., Wang, Y. & Wang, E. A comparison on predicting functional impact of genomic variants. *NAR Genom. Bioinform.* **4**, lqab122 (2022).
9. Mahmood, K. *et al.* Variant effect prediction tools assessed using independent, functional assay-based datasets: implications for discovery and diagnostics. *Hum Genomics* **11**, 10 (2017).
10. Cheng, J. *et al.* Accurate proteome-wide missense variant effect prediction with AlphaMissense. *Science* **381**, eadg7492 (2023).
11. Shihab, H. A., Gough, J., Cooper, D. N., Day, I. N. M. & Gaunt, T. R. Predicting the

functional consequences of cancer-associated amino acid substitutions. *Bioinformatics* **29**, 1504–1510 (2013).

12. Reva, B., Antipin, Y. & Sander, C. Predicting the functional impact of protein mutations: application to cancer genomics. *Nucleic Acids Res.* **39**, e118 (2011).
13. Tokheim, C. & Karchin, R. Chasmplus reveals the scope of somatic missense mutations driving human cancers. *Cell Syst.* **9**, 9-23.e8 (2019).
14. Muiños, F., Martínez-Jiménez, F., Pich, O., Gonzalez-Perez, A. & Lopez-Bigas, N. In silico saturation mutagenesis of cancer genes. *Nature* **596**, 428–432 (2021).
15. Katsonis, P., Wilhelm, K., Williams, A. & Lichtarge, O. Genome interpretation using in silico predictors of variant impact. *Hum. Genet.* **141**, 1549–1577 (2022).
16. Liu, X., Li, C., Mou, C., Dong, Y. & Tu, Y. dbNSFP v4: a comprehensive database of transcript-specific functional predictions and annotations for human nonsynonymous and splice-site SNVs. *Genome Med.* **12**, 103 (2020).
17. Frazer, J. *et al.* Disease variant prediction with deep generative models of evolutionary data. *Nature* **599**, 91–95 (2021).
18. Zhang, C., Zhang, X., Freddolino, P. L. & Zhang, Y. BioLiP2: an updated structure database for biologically relevant ligand-protein interactions. *Nucleic Acids Res.* **52**, D404–D412 (2024).
19. Chen, Y. C., Chen, Y.-H., Wright, J. D. & Lim, C. PPI-HotspotDB: Database of Protein-Protein Interaction Hot Spots. *J. Chem. Inf. Model.* **62**, 1052–1060 (2022).
20. Sanchez-Vega, F. *et al.* Oncogenic signaling pathways in the cancer genome atlas. *Cell* **173**, 321-337.e10 (2018).
21. Tunes da Silva, G., Logan, B. R. & Klein, J. P. Methods for equivalence and noninferiority testing. *Biol. Blood Marrow Transplant.* **15**, 120–127 (2009).
