# Supplementary material for: AI cancer driver mutation predictions are valid in real-world data
Source: Nat Commun. 2025 Sep 26;16:8509. doi: 10.1038/s41467-025-63461-8 (PMC12474978; doi:10.1038/s41467-025-63461-8)
Supplement: Supplementary file 3 — Description of Additional Supplementary Files [file 41467_2025_63461_MOESM3_ESM.pdf]

<u>Description of Additional Supplementary Files</u>

Supplementary Data 1. RTK/RAS mutational pattern and TMB-H status of patients with *ERBB4* or *IRS2* VUSs

Supplementary Data 2. Number of patients in GENIE v14-public with each gene sequenced
