# Supplementary material for: AI cancer driver mutation predictions are valid in real-world data
Source: Nat Commun. 2025 Sep 26;16:8509. doi: 10.1038/s41467-025-63461-8 (PMC12474978; doi:10.1038/s41467-025-63461-8)
Supplement: Supplementary file 6 — Reporting Summary [file 41467_2025_63461_MOESM6_ESM.pdf]

Corresponding author(s): Nikolaus Schultz, Justin Jee

Last updated by author(s): 8/13/2025

Reporting Summary

Nature Portfolio wishes to improve the reproducibility of the work that we publish. This form provides structure for consistency and transparency in reporting. For further information on Nature Portfolio policies, see our Editorial Policies and the Editorial Policy Checklist.

Statistics

For all statistical analyses, confirm that the following items are present in the figure legend, table legend, main text, or Methods section.

n/a	Confirmed	
☐	☒	The exact sample size (*n*) for each experimental group/condition, given as a discrete number and unit of measurement
☐	☒	A statement on whether measurements were taken from distinct samples or whether the same sample was measured repeatedly
☐	☒	The statistical test(s) used AND whether they are one- or two-sided<br>*Only common tests should be described solely by name; describe more complex techniques in the Methods section.*
☐	☒	A description of all covariates tested
☐	☒	A description of any assumptions or corrections, such as tests of normality and adjustment for multiple comparisons
☐	☒	A full description of the statistical parameters including central tendency (e.g. means) or other basic estimates (e.g. regression coefficient) AND variation (e.g. standard deviation) or associated estimates of uncertainty (e.g. confidence intervals)
☐	☒	For null hypothesis testing, the test statistic (e.g. *F*, *t*, *r*) with confidence intervals, effect sizes, degrees of freedom and *P* value noted<br>*Give P values as exact values whenever suitable.*
☒	☐	For Bayesian analysis, information on the choice of priors and Markov chain Monte Carlo settings
☒	☐	For hierarchical and complex designs, identification of the appropriate level for tests and full reporting of outcomes
☐	☒	Estimates of effect sizes (e.g. Cohen's *d*, Pearson's *r*), indicating how they were calculated

Our web collection on statistics for biologists contains articles on many of the points above.

Software and code

Policy information about availability of computer code

Data collection	R codes used in the analysis of this paper are available under MIT license on GitHub at https://github.com/clinical-data-mining/variant-annotation. Additionally, a code container is available on Zenodo at https://doi.org/10.5281/zenodo.1542105559.
Data analysis	R codes used in the analysis of this paper are available under MIT license on GitHub at https://github.com/clinical-data-mining/variant-annotation. Additionally, a code container is available on Zenodo at https://doi.org/10.5281/zenodo.1542105559.

For manuscripts utilizing custom algorithms or software that are central to the research but not yet described in published literature, software must be made available to editors and reviewers. We strongly encourage code deposition in a community repository (e.g. GitHub). See the Nature Portfolio guidelines for submitting code & software for further information.

Data

Policy information about availability of data

All manuscripts must include a data availability statement. This statement should provide the following information, where applicable:

- Accession codes, unique identifiers, or web links for publicly available datasets
- A description of any restrictions on data availability
- For clinical datasets or third party data, please ensure that the statement adheres to our policy

Source data are provided with this paper. The processed AACR Project GENIE v14 data is available on Synapse at https://www.synapse.org/Synapse:syn7222066. GENIE BPC NSCLC genomic and clinical data are available on Synapse at https://www.synapse.org/Synapse:syn27056172/wiki/616601. The MSK-IMPACT NSCLC

genomic data is available on cBioPortal as part of the MSK-CHORD cohort at https://www.cbioportal.org/study/summary?id=msk_chord_2024. All clinical annotations used for analyses in this paper are available at https://github.com/clinical-data-mining/variant-annotation to enable others to reproduce our findings and make additional discoveries. The raw sequencing data for the AACR Project GENIE and MSK-IMPACT cohorts are protected and are not broadly available due to privacy restrictions and must be requested with appropriate institutional approvals.

Research involving human participants, their data, or biological material

Policy information about studies with human participants or human data. See also policy information about sex, gender (identity/presentation), and sexual orientation and race, ethnicity and racism.

Reporting on sex and gender	Sex was retrieved from the electronic health records database and used as a covariate in inverse probability of treatment matching prior to survival analysis. No subgroup analysis was done based on sex.
Reporting on race, ethnicity, or other socially relevant groupings	Race was retrieved from the electronic health records database and used as a covariate in inverse probability of treatment matching prior to survival analysis. No subgroup analysis was done based on race.
Population characteristics	Population characteristics of the MSK-IMPACT patients with non-small cell lung cancer are described in Supplementary Table S6.
Recruitment	MSK patients were enrolled as part of a prospective sequencing protocol (NCT01775072) or analyzed as part of a IRB-approved retrospective research protocols (MSK IRB Protocols 16-1463 and 19-368). Patients provided written, informed consent and were enrolled in a continuous, nonrandom fashion.
Ethics oversight	Institutional Review Board of Memorial Sloan Kettering Cancer Center

Note that full information on the approval of the study protocol must also be provided in the manuscript.

Field-specific reporting

Please select the one below that is the best fit for your research. If you are not sure, read the appropriate sections before making your selection.

☒ Life sciences ☐ Behavioural & social sciences ☐ Ecological, evolutionary & environmental sciences

For a reference copy of the document with all sections, see nature.com/documents/nr-reporting-summary-flat.pdf

Life sciences study design

All studies must disclose on these points even when the disclosure is negative.

Sample size	No statistical methods were used to predetermine sample sizes. All available samples that met predefined inclusion criteria and were relevant to the analysis objectives were included.
Data exclusions	Data were excluded from analyses based on pre-established criteria. In the MSK-IMPACT cohort, only patients who provided informed consent and underwent tumor genomic sequencing with matched white blood cell analysis as part of a prospective protocol (NCT01775072) were included. In the AACR GENIE cohort, patients were included in gene-level analyses only if their sequencing panels covered the gene of interest. For the GENIE Biopharma Collaborative (BPC) subset, only patients with single-primary non-small cell lung cancer from three contributing institutions were included.
Replication	We have replicated primary findings in our MSK-IMPACT NSCLC cohort in a non-overlapping cohort of GENIE BPC NSCLC patients from other institutions. Some findings cannot be replicated due to the smaller cohort size of the GENIE BPC cohort, leading to insufficient number of mutations or mutated subgroups to be tested for significance. For results where replication was possible with sufficient cohort size in both MSK-IMPACT and GENIE BPC cohorts, all were replicated.
Randomization	Randomization was not relevant to this study. Demographic and clinical covariates were controlled using inverse probability of treatment weighting (IPTW), a propensity score-based method that balances covariates across comparison groups. This approach allows for adjustment of potential confounders in observational data and enables more accurate estimation of associations between genomic features and clinical outcomes.
Blinding	Blinding was not relevant to this study, as all analyses were conducted retrospectively using existing genomic and clinical data. There was no intervention or group allocation assigned by investigators.

Reporting for specific materials, systems and methods

We require information from authors about some types of materials, experimental systems and methods used in many studies. Here, indicate whether each material, system or method listed is relevant to your study. If you are not sure if a list item applies to your research, read the appropriate section before selecting a response.

Materials & experimental systems

n/a	Involved in the study
☒	☐ Antibodies
☒	☐ Eukaryotic cell lines
☒	☐ Palaeontology and archaeology
☒	☐ Animals and other organisms
☐	☒ Clinical data
☒	☐ Dual use research of concern
☒	☐ Plants

Methods

n/a	Involved in the study
☒	☐ ChIP-seq
☒	☐ Flow cytometry
☒	☐ MRI-based neuroimaging

Clinical data

Policy information about clinical studies
All manuscripts should comply with the ICMJE guidelines for publication of clinical research and a completed CONSORT checklist must be included with all submissions.

Clinical trial registration	NCT01775072
Study protocol	Protocol available from MSK (internal IRB number 12-245)
Data collection	Data here is from patients with non-small cell lung cancer at Memorial Sloan Kettering from a February 15, 2023 snapshot.
Outcomes	Primary outcome included overall survival and was obtained as described in the manuscript.

Plants

Seed stocks	*Report on the source of all seed stocks or other plant material used. If applicable, state the seed stock centre and catalogue number. If plant specimens were collected from the field, describe the collection location, date and sampling procedures.*
Novel plant genotypes	*Describe the methods by which all novel plant genotypes were produced. This includes those generated by transgenic approaches, gene editing, chemical/radiation-based mutagenesis and hybridization. For transgenic lines, describe the transformation method, the number of independent lines analyzed and the generation upon which experiments were performed. For gene-edited lines, describe the editor used, the endogenous sequence targeted for editing, the targeting guide RNA sequence (if applicable) and how the editor was applied.*
Authentication	*Describe any authentication procedures for each seed stock used or novel genotype generated. Describe any experiments used to assess the effect of a mutation and, where applicable, how potential secondary effects (e.g. second site T-DNA insertions, mosiacism, off-target gene editing) were examined.*
